# Supplementary material for: In Vivo and In Vitro Pro-Fibrotic Response of Lung-Resident Mesenchymal Stem Cells from Patients with Idiopathic Pulmonary Fibrosis
Source: Cells. 2024 Jan 16;13(2):160. doi: 10.3390/cells13020160 (PMC10814068; doi:10.3390/cells13020160)
Supplement: Supplementary file 1 [file cells-13-00160-s001.zip › cells-2770600-supplementary.pdf]

Table S1: Primer sequences.

Gene	Organism	Forward primer	Reverse primer
Col1a1	*Mus musculus*	CCAAGAAGACATCCCTGAAGTCA	TGCACGTCATCGCACACA
Fn1	*Mus musculus*	GGTGTACAACTTCCAATTACG	GGAATTTCCGCCTCGAGTCT
Il6	*Mus musculus*	ACTTCACAAGTCCGGAGAGG	TGCCATTGCACAACTCTTTTC
Tnfa	*Mus musculus*	CGGTCCCCAAAGGGATGAGAAGT	ATGATCTGAGTGTGAGGGTCTGGG
Mcp1	*Mus musculus*	CCAGCCTACTCATTGGGATCA	CTTCTGGGCCTGCTGTTCA
Hprt	*Mus musculus*	CCTAAGATGAGCGCAAGTTGAA	CCACAGGACTAGAACACCTGCTAA
Gapdh	*Mus musculus*	GAGCCCTTCCACAATGCCAAAGTT	TGTGATGGGTGTGAACCACGAGAA
TGFA	*Homo sapiens*	CTGCTGTGGCTACTGGTGC	CTTCACCAGCTCCATGTCCAT
ACTA2	*Homo sapiens*	AGAGCTACATAACACAGTTTCTCCTTGA	CGAGATCTCACTGACTACCTCATG
COL1A1	*Homo sapiens*	CAAGAGGAAGGCCAAGTCGAC	TTGTCGCAGACGCAGCAGATCC
FN1	*Homo sapiens*	GGATGTGTGGCAGATAGGATGTATT	CAATGCGGTACATGACCCCT
TNC	*Homo sapiens*	TCGGTAGCCATCCAGGAGAG	CCATCTATGGCGTGATCCGG
B2M	*Homo sapiens*	ATATTCAAACCTCCATGATGC	ACCCCCACTGAAAAAGATGAG

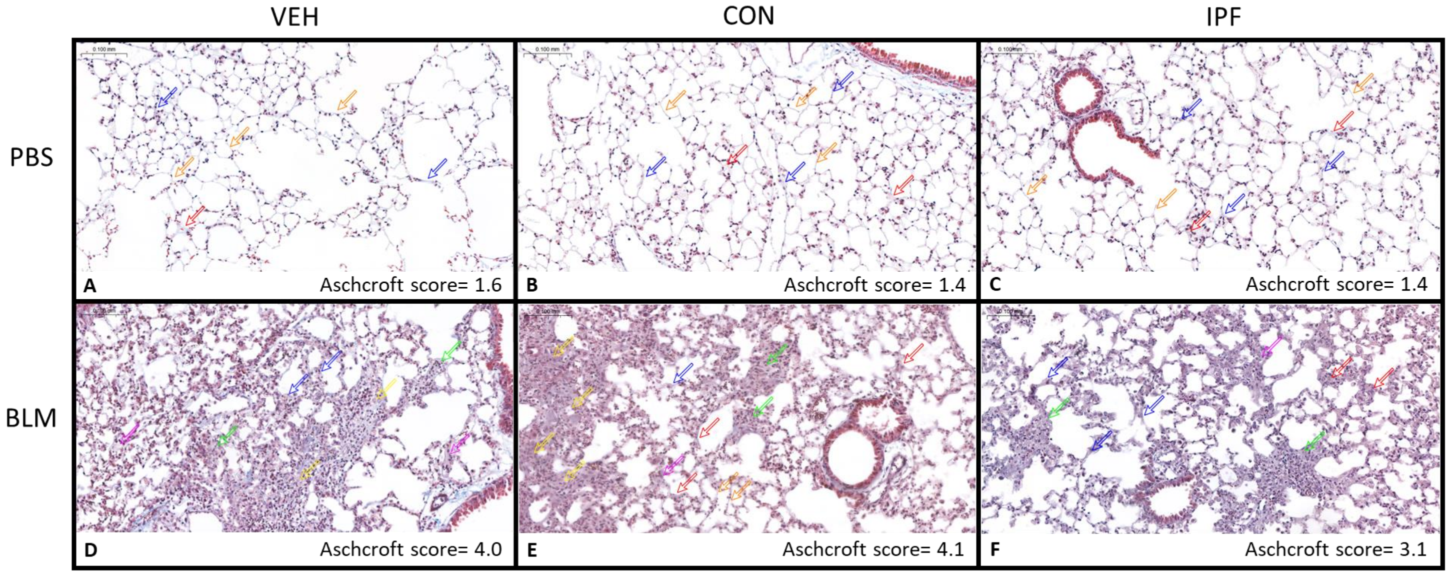

Figure S1. Histological evaluation of lung damage. Representative images of Masson's thricrome staining of one representative sample for each experimental group (PBS+VEH, PBS+CON, PBS+IPF, BLM+VEH, BLM+CON, BLM+IPF). Images were obtained at 20×. The mean Ashcroft score assessed by the modified Ashcroft scale of the selected samples is indicated. Coloured arrows indicate the grade of fibrosis in the selected field: orange, grade 0; blue, grade 1; red, grade 2; pink, grade 3; green, grade 4; yellow, grade 5. BLM: bleomycin, PBS: phosphate-buffered saline, VEH: vehicle, CON: control, IPF: idiopathic pulmonary fibrosis.

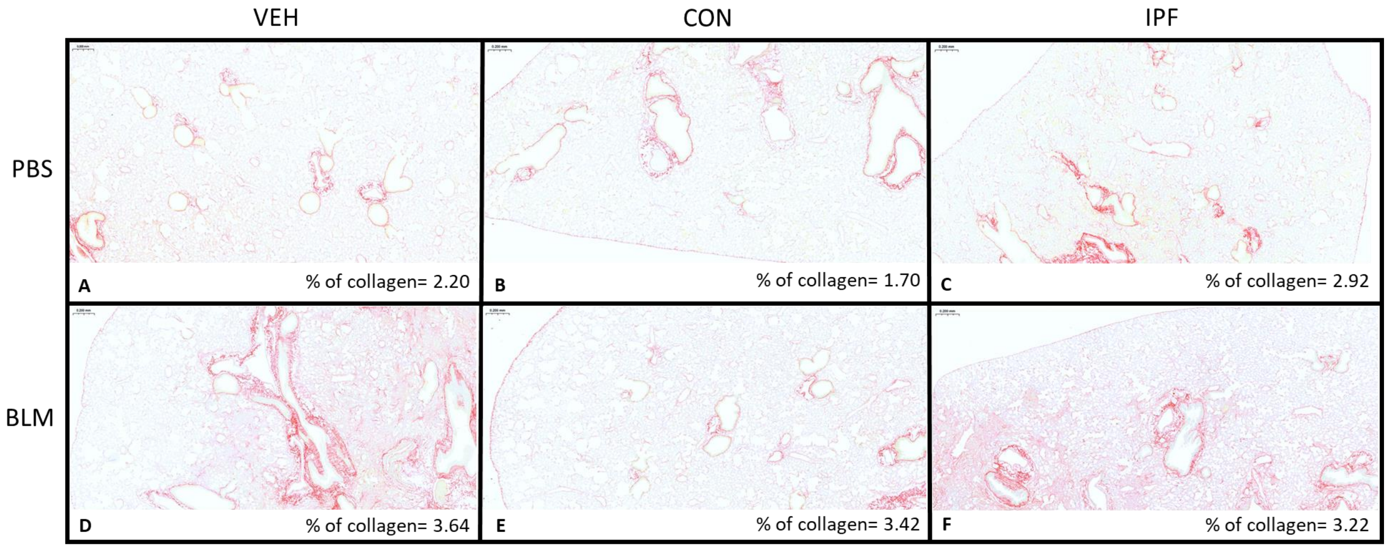

Figure S2. Histological assessment of lung collagen content. Representative images of Pricosirious Red staining of one representative sample for each experimental group (PBS+VEH, PBS+CON, PBS+IPF, BLM+VEH, BLM+CON, BLM+IPF). Images were obtained at 5x. The amount of collagen of the selected samples is indicated. Collagen quantification was expressed relative to the total lung area. BLM: bleomycin, PBS: phosphate-buffered saline, VEH: vehicle, CON: control, IPF: idiopathic pulmonary fibrosis.
